# HIV-1 Vif inhibits G to A hypermutations catalyzed by virus-encapsidated APOBEC3G to maintain HIV-1 infectivity

**DOI:** 10.1186/s12977-014-0089-5

**Published:** 2014-10-11

**Authors:** Yudi Wang, Ballington L Kinlock, Qiujia Shao, Tiffany M Turner, Bindong Liu

**Affiliations:** Center for AIDS Health Disparities Research, 1005 Dr. D. B. Todd Blvd, Nashville, Tennessee 37208 USA; Department of Microbiology and Immunology, Meharry Medical College, 1005 Dr. D. B. Todd Blvd, Nashville, Tennessee 37208 USA

**Keywords:** HIV-1, Vif, APOBEC3G, Hypermutation, Cytidine deaminase

## Abstract

**Background:**

HIV-1 viral infectivity factor (Vif) is an essential accessory protein for HIV-1 replication. The predominant function of Vif is to counteract Apolipoprotein B mRNA-editing enzyme-catalytic polypeptide-like 3G (APOBEC3G, A3G), a potent host restriction factor that inhibits HIV-1 replication. Vif mediates the proteasomal degradation of A3G and inhibits A3G translation, thus diminishing the pool of A3G that is available to be packaged into budding virion. Although Vif is robust in degrading A3G, the protection provided against A3G is not absolute. Clinical and laboratory evidence have shown that A3G is not completely excluded from HIV-1 viral particles during HIV-1 replication. It remains unclear why the viral samples are still infectious when A3G has been packaged into the virions.

**Results:**

In this study, we provide evidence that Vif continues to protect HIV-1 from the deleterious effects of A3G, even after packaging of A3G has occurred. When equal amounts of A3G were packaged into budding virions, the virus expressing functional Vif was more infectious and incurred fewer G to A hypermutations in the second round of infection compared to Vif-deficient virus. A Vif mutant with a defect in viral packaging showed a reduced ability to protect the HIV-1 genome from G to A hypermutations.

**Conclusion:**

Our data suggest that even packaged A3G is still under the tyranny of Vif. Our work brings to light an additional caveat for any therapy that hopes to exploit the Vif-A3G axis. The ideal strategy would not only enhance A3G viral packaging, but also reduce HIV-1 Vif viral encapsidation.

## Background

Apolipoprotein B mRNA-editing enzyme-catalytic polypeptide-like 3G (APOBEC3G, A3G) is a member of the APOBEC3 family of cytidine deaminases, which includes A3A, B, C, D/E and F, with A3G being the most potent against HIV-1 [1]. In the absence of Vif, A3G will encapsidate into HIV-1 virions and induce G to A hypermutations in the newly synthesized viral DNA [2-6]. A3G also exerts inhibitory effects at several other steps of HIV-1 replication, such as reverse transcription and viral DNA integration [1].

HIV-1 Vif is a 23 kDa accessory protein of HIV-1. The critical role of Vif in HIV-1 infectivity was observed shortly after the discovery of HIV-1 [7,8]; however, the mechanism by which Vif protects the integrity of HIV-1 took over a decade to decode. Seminal work by Sheehy *et al.* led to the discovery that the predominant function of Vif is to counteract A3G [9]. The general consensus is that Vif orchestrates proteasomal degradation of A3G, thus preventing its packaging into the budding virion [10-16]. During this process, HIV-1 Vif interacts with Cullin 5, A3G and the newly identified T cell differentiation factor, CBfβ, to promote formation of the Cullin 5-Vif- A3G ubiquitin E3 ligase complex, which marks A3G for proteasomal degradation [16-21]. While Vif-mediated degradation of A3G is the well-recognized mechanism by which Vif rescues HIV-1, it has also been proposed that Vif prevents A3G viral packaging through inhibiting A3G translation [15,22] or another unknown mechanism [23].

In addition to inhibiting A3G packaging, it has also been shown that Vif has the capability of directly inhibiting A3G cytidine deaminase activity in a degradation-independent manner. Santa-Marta *et al.* showed that A3G-induced cytidine deamination is inhibited by the expression of Vif, without the depletion of a deaminase domain, in an *Escherichia coli* system. Moreover, inhibition of deaminase-mediated bacterial hypermutation is dependent on a single amino acid substitution D128K that renders A3G resistant to Vif inhibition [24]. Britan-Rosich *et al.* also showed that Vif is able to inhibit A3G cytidine deaminase activity *in vitro* [25]. Recently, Feng *et al.* reported that HIV-1 Vif alters processive single-stranded DNA scanning of A3G *in vitro* [26]. However, it remains unclear if the inhibitory effect of Vif on A3G cytidine deaminase activity is integral for successful HIV-1 replication following Vif-A3G encapsidation into progeny virions. In this study, we provide evidence that Vif continues to protect HIV-1 from the deleterious effects of A3G even after packaging of A3G has occurred. When equal amounts of A3G were packaged into budding virions with functional or non-functional Vif, the virus expressing functional Vif was more infectious, and fewer G to A hypermutations were generated in the second round of infection compared to virions without functional Vif. Our work indicates that Vif plays an additional role in protecting HIV-1 from A3G after encapsidation. This work sheds light on the fact that any therapy that hopes to exploit the Vif-A3G axis needs to take into account that even though A3G becomes packaged, Vif is still able to maintain the fidelity of the virus.

## Results

### Antiviral activity of A3G is less potent against wild-type virus compared to Vif-deficient virus

**Figure 1:**
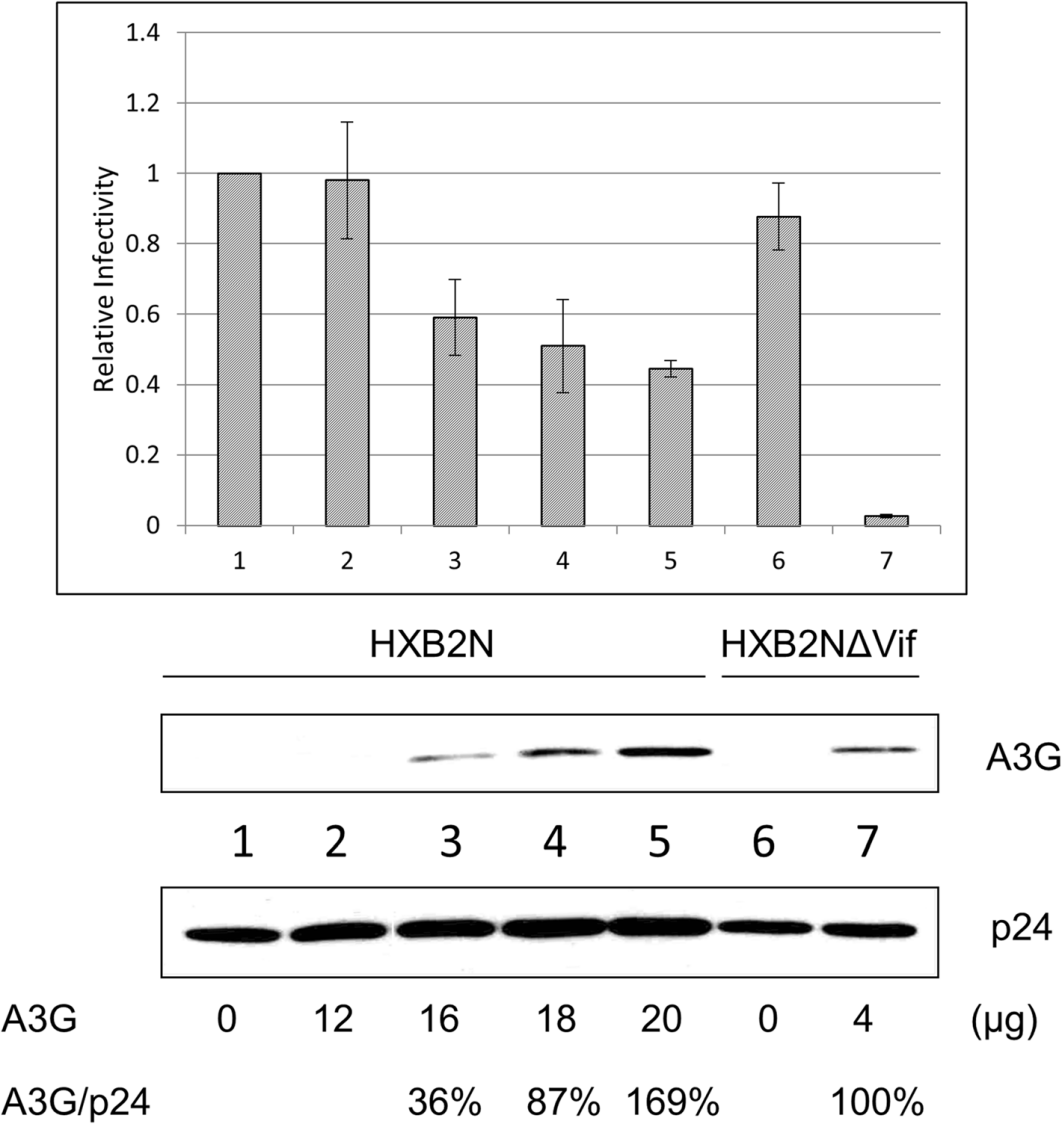
**A3G antiviral activity is circumvented more efficiently with HXB2N compared to HXB2NΔVif.** 4 μg HXB2N or HXB2NΔVif proviral construct DNA was cotransfected with indicated amount of A3G expression vector DNA into 293T cells. 4 μg pcDNA3.1 was also cotransfected with HXB2N or HXB2NΔVif into 293T cells as controls. At 48 h after transfection, cell culture supernatants were harvested and used to infect TZM-bl indicator cells to measure viral infectivity (upper panel). The concentration of viral input was normalized by p24 ELISA. Virions in cell culture supernatants were precipitated by ultracentrifugation for Western blot analysis (lower panel). The percentage represents the quantity of intraviral A3G normalized by the quantity of intraviral p24. A3G/HXB2NΔVif was set as 100%. Each experiment in this and subsequent figures was performed at least three times, and representative results are presented.

HIV-1 Vif counteracts A3G antiviral function by mediating its degradation, which leads to the exclusion of A3G from budding viral particles. However, a previous study has shown that residual amounts of A3G still exists in Vif-competent wild-type HIV-1 particles [27]. To study the antiviral function of the residual amount of A3G found in progeny virions, different amount of A3G were cotransfected with HXB2N into 293T cells. Concurrently, A3G was cotransfected with HXB2NΔVif into 293T cells. Culture supernatants were harvested from the transfected cells 48 h after transfection. The samples were purified by ultracentrifugation and subjected to MAGI assay and Western blot analysis for measuring viral infectivity and A3G expression levels in progeny virions, respectively. As shown in Figure 1, the amount of A3G in the virion from 20 μg A3G/HXB2N sample was more than 4 μg A3G/HXB2NΔVif sample (169% vs 100%). However, the infectivity of the former virus was much higher than the one of 4 μg A3G/HXB2NΔVif sample (Figure 1 lane 5 vs lane 7). These data suggest that wild-type HIV-1 circumvents A3G antiviral function more efficiently than Vif-deficient virus, and thus Vif may have an additional ability to overcome A3G even after A3G has been packaged into viral particles.

### HIV-1 Vif inhibits A3G cytidine deaminase activity in both cells and virion

**Figure 2:**
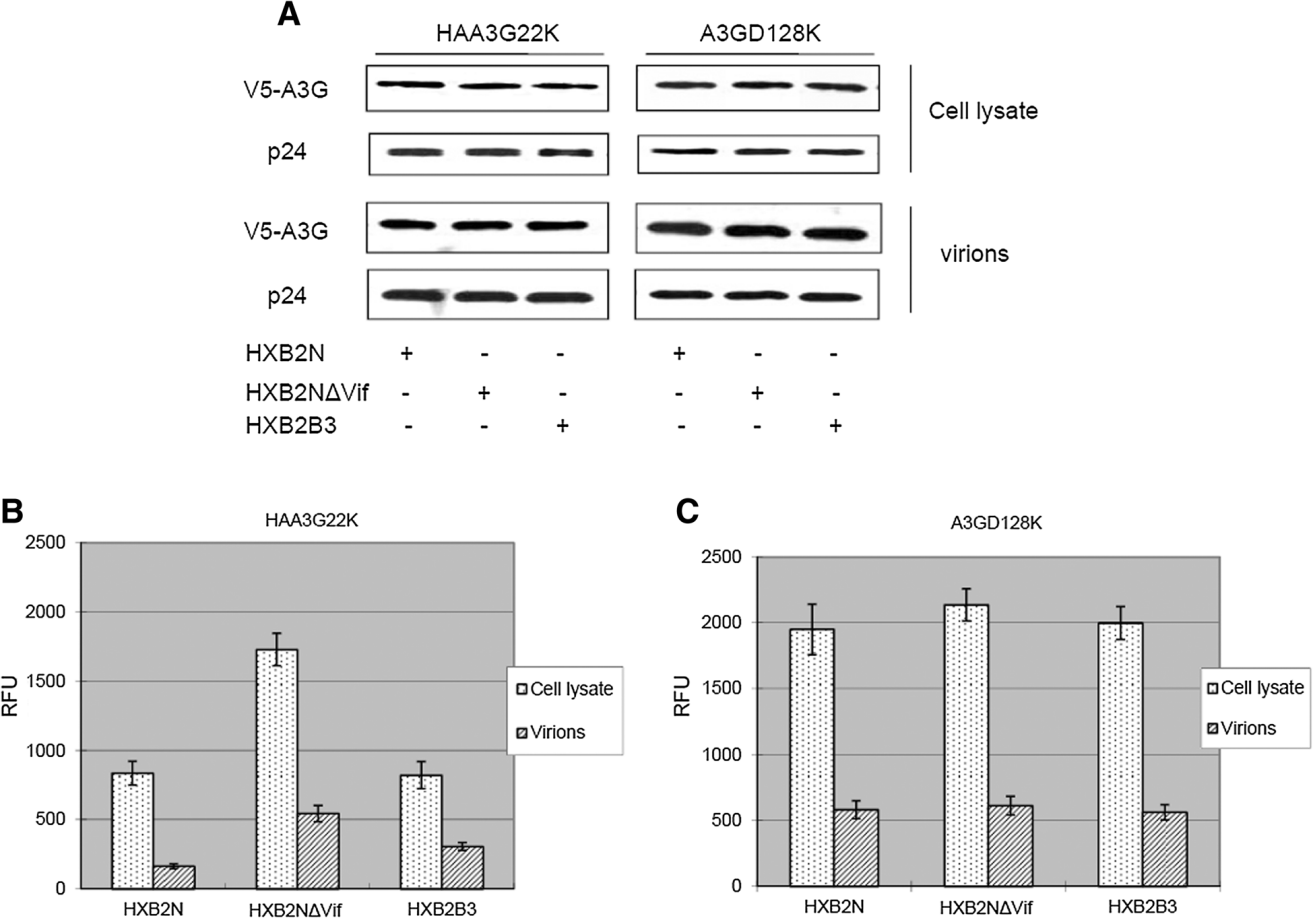
**HIV-1 Vif destroys A3G cytidine deaminase activity in both cells and virions.** 293T cells were cotransfected with HAA3G22K or A3GD128K in combination with HXB2N, HXB2NΔVif or HXB2B3. At 48 h post-transfection, the cells were collected, and virions in culture supernatants were precipitated by ultracentrifugation for Western blot analysis **(A)**. HAA3G22K was cotransfected into 293T cells with HXB2N, HXB2NΔVif or HXB2B3. At 48 h post-transfection, cells and culture supernatants were harvested to measure A3G cytidine deaminase activity **(B)**. A3GD128K was cotransfected into 293T cells with HXB2N, HXB2NΔVif or HXB2B3. At 48 h post-transfection, cells and culture supernatants were harvested to measure A3G cytidine deaminase activity **(C)**.

In our previous report, we demonstrated that a hemagglutinin (HA) tag fused to the N terminus of lysine-free A3G (HAA3G22K) renders HAA3G22K resistant to Vif-induced degradation [28]. HAA3G22K is equally packaged into wild-type and Vif-deficient HIV-1 virions. Therefore, HAA3G22K is an optimal mutant to characterize the antiviral function of A3G in the presence of HIV-1 Vif. To that end, HXB2N, HXB2NΔVif or HXB2B3 was cotransfected into 293T cells with HAA3G22K or A3GD128K. HXB2B3 contains three point mutations in the C-terminal region of Vif as described in the Methods section. Cells and culture supernatants were harvested 48 h post-transfection, and viral particles were isolated from the culture supernatants by ultracentrifugation. Cells and viral samples were subjected to the A3G cytidine deaminase assay. When Vif (Figure 2B, HXB2N) or VifB3 (Figure 2B, HXB2B3) were co-expressed with HAA3G22K in 293T cells, the cytidine deaminase activity of HAA3G22K decreased by 50% compared to Vif-deficient virus (Figure 2B, HXB2NΔVif). Even though HAA3G22K was packaged equally into the three different virions (Figure 2A), the cytidine deaminase activity of HAA3G22K was lower by 3- and 2-fold in HXB2N and HXB2B3, respectively, compared to HX-B2NΔVif (Figure 2B). These data suggest that Vif inhibits HAA3G22K cytidine deaminase activity not only in the cells, but also in the virions. When A3GD128K was used instead of HAA3G22K, there was no difference in A3GD128K cytidine deaminase activity among all three viral constructs in both the cells and virions (Figure 2C). As it has been shown that the Vif-A3G interplay is blocked when the 128 lysine residue in A3G is mutated to aspartic acid [29-31], this result (Figure 2C) suggests that the Vif-A3G interaction is essential for Vif to inhibit A3G cytidine deaminase activity.

### HIV-1 Vif reduces the G to A hypermutation rate catalyzed by A3G

**Figure 3:**
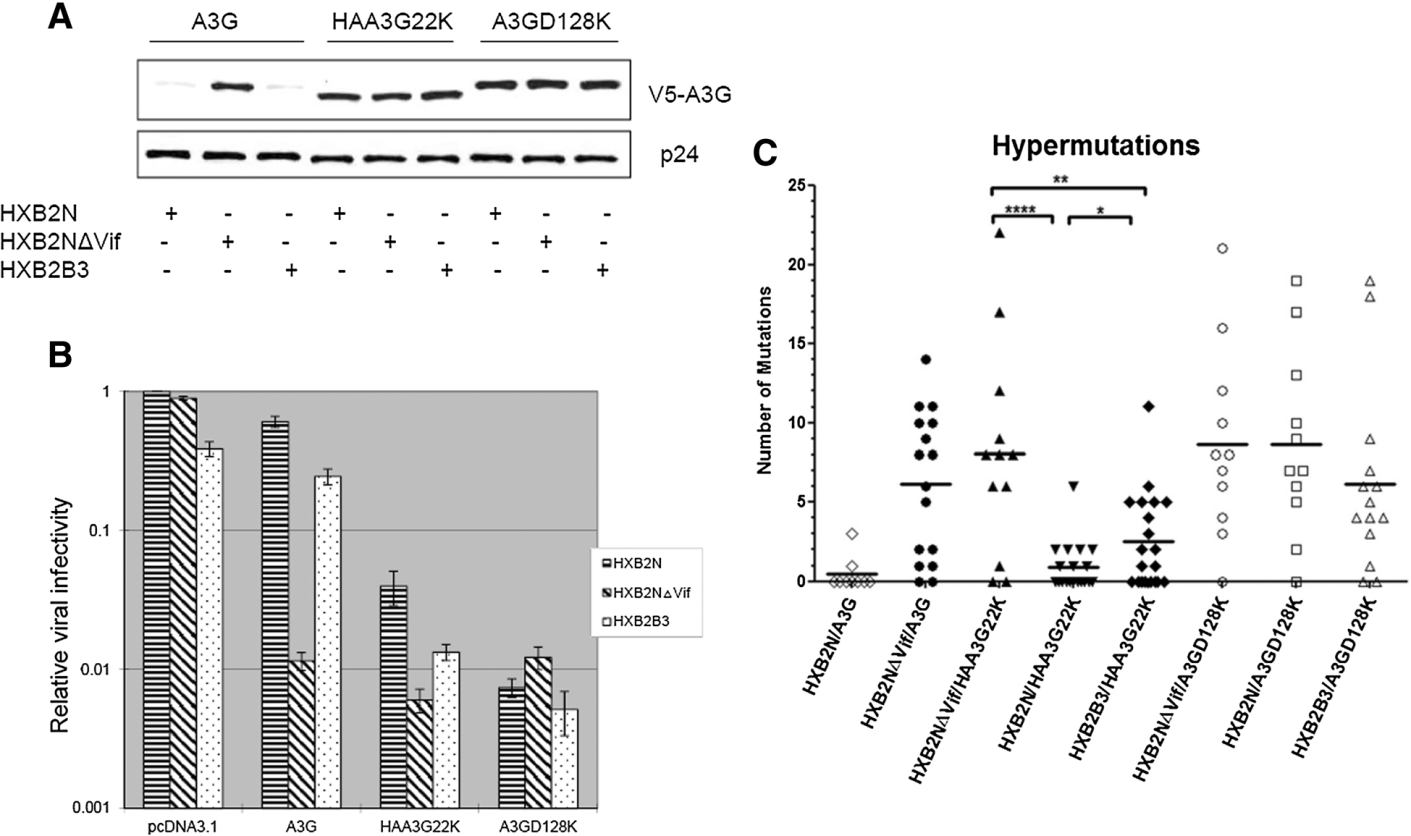
**Vif counteracts the ability of A3G to induce G to A hypermutations.** HXB2N, HXB2NΔVif or HXB2B3 was cotransfected with A3G, HAA3G22K or A3GD128K into 293T cells. At 48 h post-transfection, culture supernatants were harvested, and virus particles were precipitated by ultracentrifugation for Western blot analysis **(A)**. The cell culture supernatants were also used to infect TZM-bl indicator cells to measure viral infectivity **(B)**. The cell culture supernatants from panel A were used to infect SupT1 cells for hypermutation analysis **(C)**.

With the knowledge that Vif inhibits A3G cytidine deaminase activity in both cells and virions (Figure 2), we set out to determine if Vif interferes with the G to A hypermutation rate catalyzed by A3G. The HIV-1 proviral constructs HXB2N, HXB2NΔVif or HXB2B3 were cotransfected into 293T cells with A3G, HAA3G22K or A3GD128K. Culture supernatants were harvested 48 h post-transfection to determine the infectivity of progeny virions and to assay hypermutation levels. As shown in Figure 3A, HXB2B3 retains its capability to degrade A3G as effectively as wildtype HXB2N. Therefore the infectivity HXB2B3/pcDNA3.1 is similar to HXB2B3/A3G (Figure 3B). HAA3G22K was equally packaged into HX-B2N, HXB2NΔVif and HXB2B3 (Figure 3A). Although HAA3G22K was equally packaged, the infectivity of HXB2N/HAA3G22K was approximately 10 times higher than that of HXB2NΔVif/HAA3G22K (Figure 3B). The infectivity of HXB2B3/HAA3G22K was in the intermediate range. It is imperative to note that the only difference among these three viruses is the presence of Vif. This finding further illustrates that Vif possesses anti-A3G activity even in progeny virions. The G to A hypermutation rate catalyzed by HXB2N/HAA3G22K was significantly lower than that of HXB2NΔVif/HAA3G22K (Figure 3C), while that of HXB2B3 ranged in between those values. This result indicates that Vif reduces the G to A hypermutation rate catalyzed by A3G even after A3G is packaged into viral particles. Using the A3GD128K mutant instead of HAA3G22K showed that Vif was unable to maintain viral infectivity (Figure 3B), and the rate of G to A hypermutation (Figure 3C) was constant, further indicating that Vif requires interaction with A3G to maintain viral infectivity.

### VifB3 mutant encapsidation into HIV particles is defective

**Figure 4:**
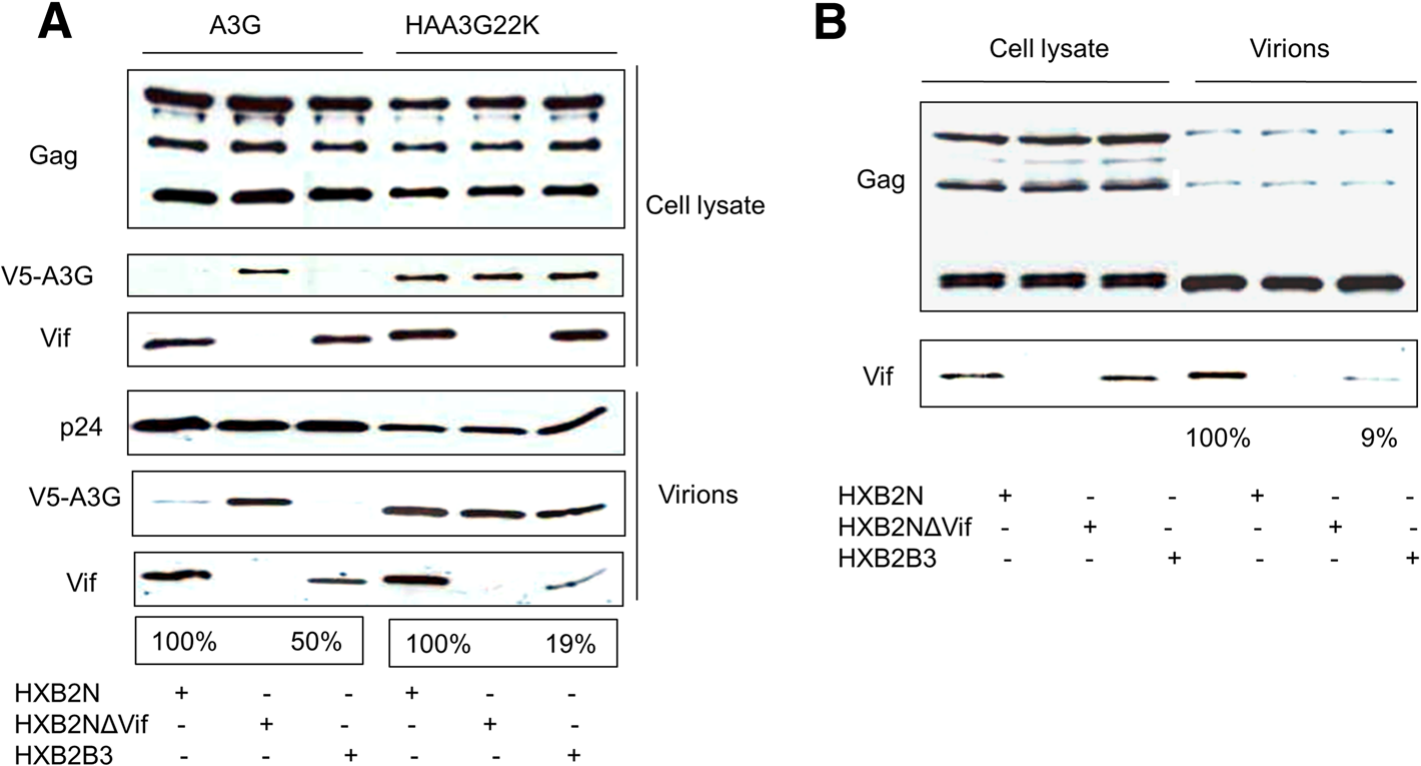
**VifB3 mutant encapsidation into HIV particles is defective.** 293T cells were cotransfected with A3G or HAA3G22K and a proviral construct HXB2N, HXB2NΔVif or HXB2B3. At 48 h post-transfection, cells and culture supernatants were harvested, and viral particles were precipitated by ultracentrifugation for Western blot analysis **(A)**. HXB2N, HXB2NΔVif or HXB2B3 was transfected into 293T cells. At 48 h post-transfection, cells were collected, and viral particles in culture supernatants were precipitated by ultracentrifugation for Western blot analysis **(B)**. The percentage represents the quantity of intraviral Vif normalized by the quantity of intraviral p24. HXB2N was set to 100%.

Compared to wild-type Vif, VifB3 is less efficient in inhibiting A3G cytidine deaminase activity in virions (Figure 2B), reducing G to A hypermutations (Figure 3C) and retaining viral infectivity (Figure 3B, HAA3G22K). The viral replication of the HXB2B3 mutant has been reported to be similar to that of wild-type HIV-1 virus in SupT1 cells. However, the HXB2B3 mutant virus shows delayed replication in H9 cells compared to wild-type virus [32]. Unlike the high levels of A3G found in H9 cells, SupT1 cells express negligible to no A3G. From these observations, we considered that the VifB3 mutant may have a defect in overcoming A3G antiviral activity compared to wild-type Vif. To test this hypothesis, we transfected HXB2N, HXB2NΔVif or HXB2B3 with A3G or HAA3G22K into 293T cells. Cells and viral samples were analyzed by Western blot. Both HXB2N and HXB2B3 efficiently induced the degradation of A3G in cells and excluded A3G from viral particles (Figure 4A). As expected, HAA3G22K was resistant to HXB2N- and HXB2B3-induced degradation (Figure 4A). Surprisingly, the HXB2B3 virus packaged less Vif compared to wild-type HXB2N virus despite equal expression levels found in the cells (Figure 4A). Similar results were also obtained in the absence of A3G or HAA3G22K, which suggests that VifB3 packaging is independent of A3G and HAA3G22K (Figure 4B). Taken together (Figures 2 and 3), these data show that packaging of Vif is essential for it to inhibit A3G cytidine deaminase activity in virions, reduce the G to A hypermutation rate and retain HIV-1 infectivity. Less VifB3 than wild-type Vif was packaged into budding virions, which may explain why the activity of VifB3 always ranged between that of wild-type Vif and no Vif (i.e., Vif-deficient virus) (Figures 2 and 3).

### Vif retains infectivity but HIV-1 replication is delayed in HAA3G22K-expressing Jurkat cells

**Figure 5:**
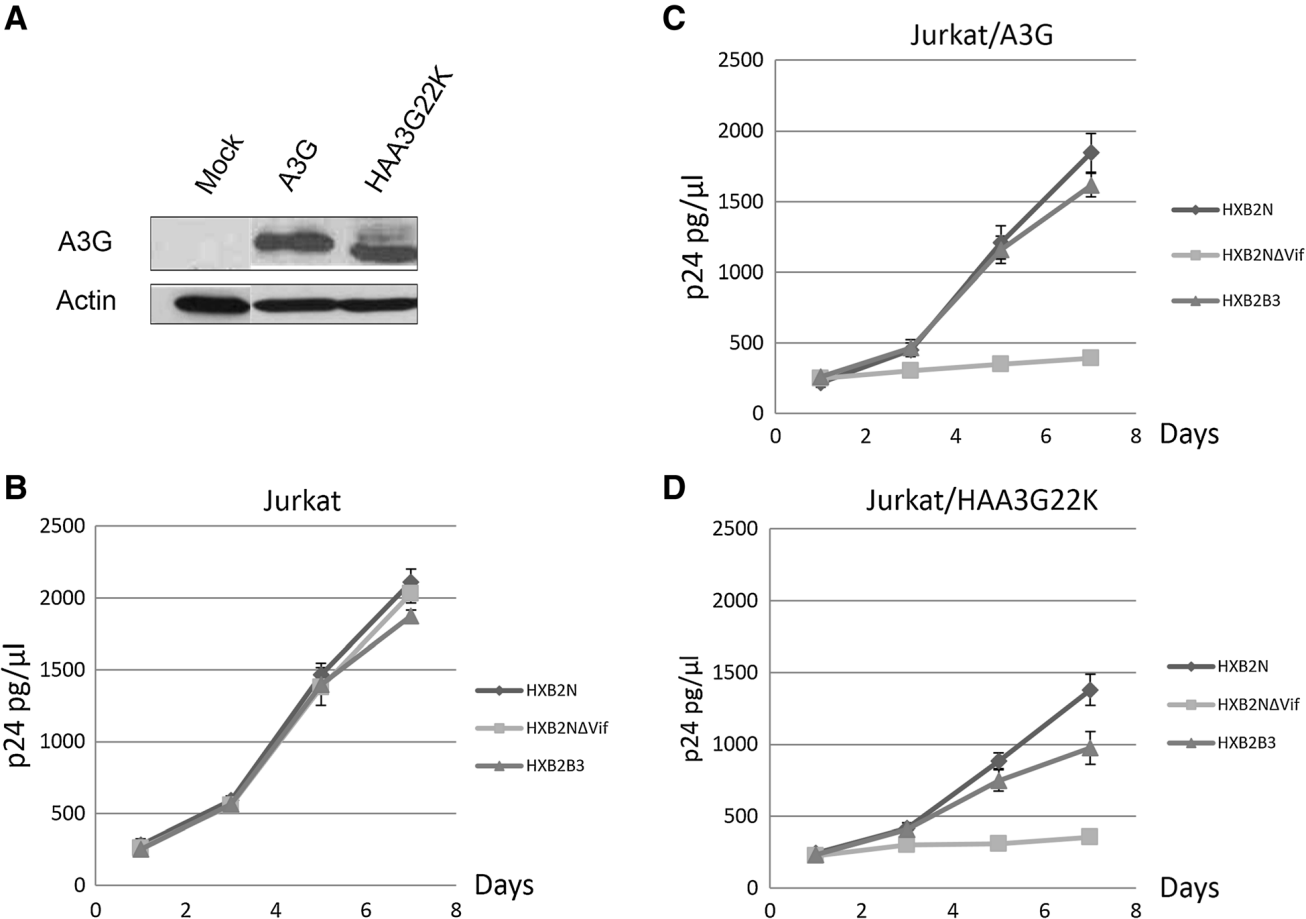
**HXB2N virus replicates more efficiently than HXB2NΔVif or HXB2B3 in Jurkat cell lines stably expressing HAA3G22K.** Jurkat cells stably expressing A3G or HAA3G22K were analyzed by Western blot **(A)**. Virus derived from HXB2N, HXB2NΔVif or HXB2B3 was used to infect Jurkat **(B)**, Jurkat/A3G **(C)** and Jurkat/HAA3G22K **(D)** stable cell lines. Viral supernatants were harvested on days 1, 3, 5 and 7 post-infection and analyzed by p24 ELISA to measure HIV-1 levels.

We have shown that Vif partially retains HIV-1 infectivity even after HAA3G22K was packaged into the budding virion (Figure 3B). To further explore this phenomenon in a T cell system, we established Jurkat cell lines stably expressing A3G or HAA3G22K. Western blot analysis showed that similar levels of A3G and HAA3G22K were expressed in the selected cell line (Figure 5A). HXB2N, HXB2NΔVif or HXB2B3 virus was used to infect Jurkat, Jurkat/A3G and Jurkat/HAA3G22K cell lines. As expected, all three viruses replicated very well in Jurkat cell line (Figure 5B), while HXB2NΔVif could not replicate in the Jurkat/A3G (Figure 5C) and Jurkat/HAA3G22K (Figure 5D) cell lines. Wild-type HXB2N virus only partially replicated in the Jurkat/HAA3G22K cell line (Figure 5D) compared to its replication in Jurkat (Figure 5B) and Jurkat/A3G (Figure 5C) cell lines. These results indicate that Vif was able to partially maintain HXB2N viral infectivity even after HAA3G22K was packaged into HXB2N virions. In addition, the replication of HXB2B3 was determined not to be robust as HXB2N due to reduced packaging of VifB3.

## Discussion

The discovery of A3G as a potent anti-HIV-1 host restriction factor has fostered interest in blocking the Vif-A3G interaction and ultimately inhibiting Vif-mediated A3G degradation as a potential treatment strategy. Vif drastically reduces the cellular pool of A3G and consequently prevents A3G from hitch-hiking a ride with the nucleocapsid of Gag, viral RNA or 7SL RNA into the budding virion, thus preserving HIV-1 infectivity [33]. Several studies have also shown that Vif prevents A3G viral packaging through inhibiting A3G translation [15,22] or other mechanisms [23].

A3G inhibits HIV-1 replication at several steps of the HIV-1 life cycle, such as reverse transcription and viral cDNA integration. However, the hallmark of the potent antiviral phenotype of A3G is found in its ability to induce lethal G to A hypermutations in the viral genome. A3G has a strong bias towards catalyzing hypermutations on minus strand DNA 5’ CC or 5’ CCC. These mutations may lead to the introduction of premature stop codons and/or result in non-infectious or defective proviruses [34]. Although Vif is robust in degrading A3G, thus maintaining viral infectivity, the protection provided against A3G is not absolute. This notion is evident by the G to A hypermutations found in genomes of primary viruses isolated from AIDS patients [35-38]. The dominant mutation patterns found among those hypermutations were in the GG to AG context, which suggests that these mutations were induced by A3G. These findings illustrate that A3G is not completely excluded from HIV-1 viral particles even in the presence of Vif. Observations by Gillick *et al.* suggest that detectable amounts of A3G are present in wild-type HIV-1 particles produced from CD4^+^ T cells during the course of infection [27]. However, Xu *et al*. reported that virion incorporation of approximately seven A3G molecules is sufficient to inhibit the replication of Vif-deficient HIV-1 [39]. The obvious discrepancy is that the primary isolates from viral samples were Vif-competent virus, whereas the latter study involved Vif-deficient virus. Taken together, these studies give credence to the argument that Vif may preserve HIV-1 infectivity even after A3G has been packaged into the virion, and our results are consistent with this idea. As shown in Figure 1, even though more A3G was incorporated into Vif-competent HIV (sample 5) compared to Vif-incompetent HIV (sample 7), the infectivity of Vif-competent HIV was much higher than the one of Vif –incompetent HIV. Furthermore, when HAA3G22K, the A3G mutant resistant to Vif-mediated degradation, was used to ensure equal packaging of A3G into both wild-type and Vif-deficient viruses, the infectivity of wild-type virus was 10-fold greater than that of Vif-deficient virus. This result corresponds to the G to A hypermutation rate of wild-type virus, which was significantly lower than that of Vif-deficient virus (Figure 3). Ultimately, our results provide evidence that Vif harbors a backup mechanism to counteract A3G antiviral function even after A3G is packaged into the budding virion.

Our lab previously demonstrated that A3G reduces early and late viral reverse transcription products equally in the presence or absence of Vif [28]. This ability of A3G may explain why the viral infectivity of wild-type virus HXB2N in the presence of HAA3G22K was reduced 10-fold compared to HXB2N with the pcDNA3.1 control vector in our system (Figure 3B). In the absence of Vif, however, A3G would further diminish the integrity of the viral genome through G to A hypermutations in the newly synthesized viral cDNA, which would not occur in a Vif-proficient virus, as shown in Figure 3C. These data suggest that G to A hypermutations further reduce the infectivity of Vif-deficient HIV-1. A3G cytidine deaminase inactive mutants (such as H257R, C288S and C291S) inhibit HIV-1 replication through reducing viral cDNA production [40]. These mutants do not generate G to A hypermutations [41]. Therefore, we do not expect Vif will have effects on the antiviral function of the intraviral form of these mutants.

## Conclusions

In summary, we used an HIV-1 replication system to show that HIV-1 Vif has alternate functions for counteracting A3G and preserving HIV-1 infectivity. While the initial function of Vif is to prevent A3G viral incorporation, this work confirms that Vif also inhibits the G to A hypermutations catalyzed by A3G. Therefore, any therapy that hopes to exploit the Vif-A3G axis must take into account the ability of Vif to overcome A3G in both the producer cell and the virion. Thus, even though A3G becomes packaged, Vif is still able to maintain the fidelity of the virus. A practical strategy for an antiviral treatment would be not only to increase A3G viral incorporation but also to reduce Vif viral packaging.

## Methods

### Plasmids, antibodies and reagents

The proviral DNA constructs of wild-type HIV-1 (HXB2N) and Vif mutant (HXB2NΔVif) as well as Vif-cMyc expression vectors were provided by Xiao-Fang Yu (Johns Hopkins University) [11,16]. HXB2B3 contains three point mutations in the C-terminal region of Vif as previously described [42]. The three point mutations were first introduced into a Vif transfer vector, which contained the EcoRI- EcoRI DNA fragment of HXB2N, using the primer GATGGAACAAGCCCCAGGCGACCGCGGGCCACGCAGGGAGCCACACAATGA and QuikChange Lightning Multi Site-Directed Mutagenesis Kit (Agilent Technologies). The DNA fragment containing the three point mutations was then sub-cloned into the HXB2N construct to generate HXB2B3. Wild-type A3G and A3GD128K mutant plasmids were gifts from Yong-Hui Zheng (Michigan State University). The Vif-resistant A3G mutant, HA-A3G20K/RΔ2K (hereafter referred to as HAA3G22K), was previously described [28]. The monoclonal anti-V5 antibody was purchased from Invitrogen. The rabbit anti-Vif antibody, rabbit anti-A3G antibody [43] and HIV-1 p24 monoclonal antibody [44] were obtained from NIH-ARRRP.

### Cell culture, HIV-1 preparation, HIV-1 purification, DNA transfection, Western blot analysis and viral infectivity (MAGI) assay

Human embryonic kidney (HEK) 293T cells and TZM-bl cells [45] (NIH-ARRRP) were cultured in DMEM containing 10% fetal bovine serum (FBS) in 5% CO_2_ atmosphere at 37°C. The culture supernatant was harvested 48 h post-transfection for the MAGI assay and viral preparation. HIV-1 virions were prepared from the cell culture supernatant and separated from cellular debris by centrifugation at 1,000 × *g* for 15 min and filtered through a 0.2 μm pore size membrane. Virus particles were concentrated by ultracentrifugation at 100,000 × *g* for 2 h on a 20% sucrose cushion. Transfections were performed using polyethylenimine (PEI). Western blot analysis and the MAGI assay were carried out as previously described [28,46].

### Hypermutation assay

293T cells were cotransfected with HIV-1 expression vectors and A3G expression vectors as indicated. Culture supernatants were collected 48 h post-transfection and treated with DNase I (20 U/ml) at 37°C for 1 h. SupT1 cells (1 × 10^6^) were spin-infected with DNase I-treated HIV-1 (150 ng p24^Gag^ equivalent) at 2000 × *g* for 2 h at room temperature. The cells were then washed with fresh medium and cultured at 37°C, 5% CO_2_ atmosphere. Cells were harvested 12 h post-infection. DNA was isolated using a DNeasy Blood and Tissue DNA isolation kit (QIAGEN). A 650-bp DNA fragment covering a portion of *nef*, U3, and R of HIV-1 was amplified with *Taq* DNA polymerase (Invitrogen) using the primers HIV-1-F (5′-AGGCAGCTGTAGATATTAGCCAC) and HIV-1-R (5′-GTATGAGGGATCTCTAGCTACCA). The PCR products were cloned into the TOPO TA-cloning vector pCR2.1 (Invitrogen). The clones were sequenced, and the sequencing results were analyzed using the CLC Main Workbench software. Statistical analysis was performed using the GraphPad Prism 5 software.

### A3G cytidine deaminase assay

The FRET-based cytidine deaminase assay was performed based on a modified protocol adapted from a previously described method [47]. 293T cells were cotransfected with the indicated HIV-1 expression vector and the indicated A3G expression vector. At 48 h post-transfection, cells and supernatant were harvested, and the virus was concentrated from the supernatant by ultracentrifugation. NP40 buffer (0.626% NP40, 10 mM Tris acetate pH 7.5, 50 mM potassium acetate, 10 mM NaCl) was used to lyse both cell and viral samples. The cell or viral lysate (15–20 μl) was mixed with 70 μl master mix containing 20 pmol Taqman positive control probe (56-FAM/TTATTATTCCCATTTGATT/-36TAMSp), 1.0 unit uracil DNA glycosylase (NEB), 50 mM Tris (pH 7.4), 10 mM EDTA, 250 μg/ml RNase A (Qiagen) and 1 μM fluorescence. Cell and viral lysate samples were incubated at 37°C for 3 h or overnight, respectively. Thereafter, 4 μl of 4 M NaOH was added to the reaction and incubated for 30 min at 37°C. The reaction was then neutralized by adding 4 μl of 4 M HCl and 36 μl of 2 M Tris (pH 7.9). The samples were cooled to 4°C, and fluorescence was measured using a MyiQ ICycler (Bio-Rad). To measure the background of each sample, the Taqman negative control probe (56-FAM/TTATTATTGGGATTTGATT/36-TAMSp) was used. The cytidine deaminase activity was calculated as a function of the activity generated from the positive probe minus the activity generated from the negative probe.

### Jurkat A3G and Jurkat HAA3G22K stably expressing cell lines and HIV-1 replication

To establish stable cell lines, Jurkat cells (1 × 10^6^) were washed twice with PBS and pulsed with the A3G or HAA3G22K plasmid (3 μg each) suspended in 10 μl Buffer R using the Invitrogen Neon Transfection System (pulse voltage 1425 V, pulse width 10 ms and pulse number 3). After electroporation, the cells were cultured in RPMI with 10% FBS for two days. G418 (1 mg/ml, Invitrogen) was added to the culture on day 3 for neomycin resistance selection. Single clones were selected using the limited dilution method. The APOBEC expression of the selected cell lines was monitored by Western blot. HXB2N, HXB2NΔVif and HXB2B3 proviral constructs were transfected into 293T cells to produce the corresponding viruses. Jurkat, Jurkat/A3G and Jurkat/HAA3G22K cells (6 × 10^5^) were spin-infected with 150 ng of p24 HXB2N, HXB2NΔVif or HXB2B3 virus. After extensive washes with RPMI, the infected Jurkat cell lines were cultured at 37°C in a 5% CO_2_ incubator, and the supernatant (200 μl) was harvested on days 1, 3, 5 and 7 post-infection. An equal volume of fresh culture media was added to the culture to maintain a constant culture volume after each harvest. HIV-1 levels were assayed using a p24 ELISA kit (AIDS and Cancer Virus Program, National Cancer Institute, Frederick, MD).
